# Dynamic Processes and Mechanisms Involved in Relaxations of Single-Chain Nano-Particle Melts

**DOI:** 10.3390/polym13142316

**Published:** 2021-07-14

**Authors:** Jon Maiz, Ester Verde-Sesto, Isabel Asenjo-Sanz, Paula Malo de Molina, Bernhard Frick, José A. Pomposo, Arantxa Arbe, Juan Colmenero

**Affiliations:** 1 Centro de Física de Materiales (CFM) (CSIC-UPV/EHU)-Materials Physics Center (MPC), Paseo Manuel de Lardizábal 5, 20018 Donostia-San Sebastián, Spain; mariaester.verde@ehu.eus (E.V.-S.); misabel_asenjo@ehu.eus (I.A.-S.); p.malodemolina@ehu.eus (P.M.d.M.); josetxo.pomposo@ehu.eus (J.A.P.); mariaaranzazu.arbe@ehu.eus (A.A.); juan.colmenero@ehu.eus (J.C.); 2 IKERBASQUE-Basque Foundation for Science, Plaza Euskadi 5, 48009 Bilbao, Spain; 3 Institut Laue-Langevin, 71 Avenue des Martyrs, CEDEX 9, 38042 Grenoble, France; frick@ill.fr; 4 Departamento de Polímeros y Materiales Avanzados: Física, Química y Tecnología, Universidad del País Vasco-Euskal Herriko Unibertsitatea (UPV/EHU), 20018 Donostia-San Sebastián, Spain; 5 Donostia International Physics Center, Paseo Manuel de Lardizábal 4, 20018 Donostia-San Sebastián, Spain

**Keywords:** single-chain nano-particles, polymer dynamics, quasielastic neutron scattering, intra-molecular cross-links

## Abstract

We present a combined study by quasielastic neutron scattering (QENS), dielectric and mechanical spectroscopy, calorimetry and wide-angle X-ray diffraction on single-chain nano-particles (SCNPs), using the corresponding linear precursor chains as reference, to elucidate the impact of internal bonds involving bulky cross-links on the properties of polymer melts. Internal cross-links do not appreciably alter local properties and fast dynamics. This is the case of the average inter-molecular distances, the β-relaxation and the extent of the atomic displacements at timescales faster than some picoseconds. Contrarily, the α-relaxation is slowed down with respect to the linear precursor, as detected by DSC, dielectric spectroscopy and QENS. QENS has also resolved broader response functions and stronger deviations from Gaussian behavior in the SCNPs melt, hinting at additional heterogeneities. The rheological properties are also clearly affected by internal cross-links. We discuss these results together with those previously reported on the deuterated counterpart samples and on SCNPs obtained through a different synthesis route to discern the effect of the nature of the cross-links on the modification of the diverse properties of the melts.

## 1. Introduction

Over the last years, new synthesis strategies have been developed in order to obtain new polymers with properties similar to those of folded biomolecules. A new family of macromolecular objects based on purely intra-molecular bonding of single polymer chains emerges as promising. Among others, the so-called single chain polymer nano-particles (SCNPs) have attracted significant attention due to their potential applications and special characteristics such as their small size, softness and internal segmentation [1,2,3,4,5,6,7]. SCNPs are unimolecular nano-objects obtained by intra-molecular cross-link of individual macromolecular chains (functionalized linear polymers called “precursors”). Bulky materials exclusively made of SCNPs are particularly interesting since they are systems half-way between polymers and colloids.

Polymer melts are complex systems where the relevant dynamic processes depend on the length scale of observation [8,9]. Understanding both “macroscopic” and “microscopic” polymer dynamics is important to design, develop, and fabricate new bulky materials. In general, physical properties (e.g., mechanical, thermal, transport properties) are influenced by the different dynamical processes taking place in polymers [10]. These polymer dynamics can be explored by different experimental methods. When the *T* >> *T_g_* (*T_g_* is the glass-transition temperature), quasielastic neutron scattering (QENS), including neutron spin echo spectroscopy (NSE), can characterize fast polymer relaxations. Through the scattering vector (*Q*) dependence of the measured magnitudes, QENS provides spatial information about the dynamical mechanisms taking place in the sample, covering an equivalent time window of approx. τ ∼ 10^−12^–10^−7^ s. Processes such as chain diffusion, reptation, Rouse dynamics or the primary α-relaxation can be observed at such high temperatures, depending on the length scale of observation [9,11]. At lower temperatures approaching the glass transition, broadband dielectric spectroscopy (BDS) covering time-scales τ ∼ 10^−7^–10^0^ s gives the opportunity to continue measuring the *α*-relaxation, where polymer segments cooperatively relax and reorient in the supercooled liquid, as well as secondary relaxations. Finally, when the segmental relaxation time is slow (τ ≳ 1 s), differential scanning calorimetry (DSC) is the most used characterization technique to determine the value of the glass-transition temperature *T_g_*. In addition, different mechanical techniques such as rheology and dynamic mechanical analysis (DMA) can be used to probe the dynamics of segments and address intermediate dynamics and terminal relaxation of the chains.

The combination of these different but complementary techniques allows also the study of the properties of SCNP melts. In recent works [12,13,14], the structure and dynamics in melts of SCNPs based on poly(tetrahydrofuran) (PTHF) synthesized via two different mechanisms have been studied applying such a methodology. The first synthesis mechanism was based on the intra-molecular azide photodecomposition process, exposed to UV irradiation procedure. The use of light/UV driven chemistry is also relevant for e.g., anthracenes [15]. The second route was via “click” chemistry, where an external homobifunctional cross-linker was used to prepare the SCNP samples. Deuterated materials were investigated, such that the neutron scattering investigation addressed collective features [12,13,14]. In an overall view, it was found that internal cross-links do not influence the average inter-molecular distances in the melt, while they have a profound impact at intermediate length scales (length scales larger than the inter-molecular ones but not yet in the hydrodynamic regime). The structural relaxation process as directly monitored by NSE at the first structure factor peak is influenced by the nature of the internal bonds used. When bulky cross-linkers are involved in the system, the structural relaxation is slowed down, while no effect is observed when the macromolecules are directly connected with covalent bonds as it is the case of the SCNPs synthesized via UV irradiation.

**Scheme 1:**
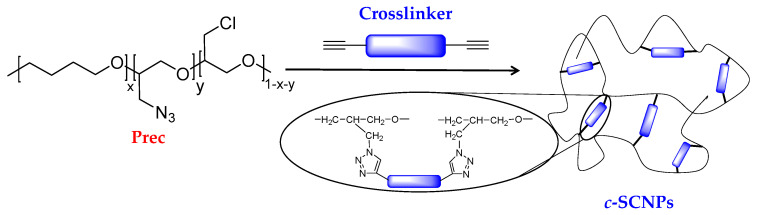
Schematic representation of the synthesis of SCNPs via “click” chemistry reaction using propargyl ether as homobifunctional cross-linker.

In this work we present a combined study by different experimental techniques, including calorimetry, mechanical analysis, dielectric spectroscopy and quasielastic neutron scattering on bulk samples consisting of protonated SCNPs synthesized from poly(tetrahydrofuran)-based linear precursors (Prec) via copper “click” chemistry mechanism, as shown in Scheme 1. Crosslinking is induced under high dilution conditions of the precursor chains in order to avoid unwanted inter-molecular bonding during the synthesis procedure. Once the SCNPs are obtained in these conditions, melts composed by them are produced. To minimize—as much as possible—potential inter-particle cross-linking in the melt state, SCNPs that have been intra-molecularly cross-linked to the maximum extent were considered in this work.

Melts of the corresponding precursor chains are investigated in parallel for comparison. The aim of the QENS experiments was to follow the self-atomic motions (hydrogen) in energy-resolved experiments to study the dynamical properties at local length scales when the chain topology is altered by internal cross-linkers. The results are compared with those obtained on the deuterated counterparts previously published [14] and with those reported for the melts of protonated SCNPs obtained via UV irradiation from the same precursor chains [13]. 

## 2. Materials and Methods

### 2.1. Materials

All chemical reagents and solvents were obtained from Sigma-Aldrich (Munich, Germany), Scharlab (Barcelona, Spain) and Eurisotop (Saint-Aubin, France). Tetrahydrofuran (THF, ≥99.9% (GC), Scharlab, Barcelona, Spain) was dried using a drying agent (calcium hydride, CaH_2_, 95%, Aldrich, Munich, Germany), degassed by three freeze-degas-thaw cycles and distillated under reduced pressure at 323 K. (±)-Epichlorohydrin (ECH, ≥99% (GC), Sigma Aldrich) was purified by distillation following the same procedure explained before for the purification of THF. The purification of tris(pentafluorophenyl)borane (B(C_6_F_5_)_3_, 95%, Sigma Aldrich) was performed by sublimation under reduced pressure at 333 K using a cold finger condenser. Dichloromethane (CH_2_Cl_2_, anhydrous, Sigma Aldrich) was degassed by bubbling Argon for 30 min prior to use. (+)-Sodium l-ascorbate (BioXtra, ≥99% (NT), Sigma Aldrich), *N*,*N*,*N*′,*N*″,*N*″-pentamethyldiethylenetriamine (PMDETA, 99%, Sigma Aldrich), Propargyl ether (98%, Sigma Aldrich), *N*,*N*-dimethylformamide (DMF, anhydrous, ≥99.8%, Scharlab), sodium azide (NaN_3_, ≥99.5%, Sigma Aldrich), and methanol (MeOH, ≥99.9%, Scharlab) were used as received. Copper (I) bromide (CuBr, 98%, extra pure, Acros Organics, Fair Lawn, NJ, USA) was purified following the Keller and Wycoff method [16].

### 2.2. Synthesis Methods

Synthesis of THF and ECH (P(THF-*co*-ECH)) copolymer: This copolymer was synthesized following the same synthetic procedure developed by our group [17]. The reactions was performed in bulk using a Schlenk flask under argon atmosphere. B(C_6_F_5_)_3_ (200 mg, 0.4 mmol), THF (25.0 mL, 308.0 mmol) and ECH (6.3 mL, 81.0 mmol) were mixed in a 100 mL Schlenk flask and stirred at room temperature (r.t.) for 48 h. The crude product of the reaction was precipitated in 600 mL of cold MeOH and dried at 333 K under vacuum for 24 h, producing a sticky transparent copolymer (P(THF-*co*-ECH): 21.5 g, 71% yield). The ECH content in P(THF-*co*-ECH) was calculated by Nuclear Magnetic Resonance (NMR) and estimated to be 27 mol%.

Synthesis of the azide-containing precursor (Prec) by azidation of the obtained copolymer P(THF-*co*-ECH): a solution of 4.0 g of P(THF-*co*-ECH) in 160 mL anhydrous DMF (160 mL) was prepared into a round-bottom flask of 500 mL. Then, NaN_3_ (1.7 g) was added into the solution, and the obtained suspension was stirred for 24 h at 333 K. The crude product was filtrated to remove NaN_3_ and evaporated under reduced pressure until get *ca* 15 mL. Then, the resulting crude was precipitated in a cold mixture of 400 mL of 1:4 H_2_O/MeOH and dried at 323 K under vacuum for 24 h to obtain the precursor (Prec: 6.8 g, 87% yield). The molecular weight *M_w_* was 22 kg/mol with polydispersity *Ð* = *M_w_*/*M_n_* of 1.24 (determined by Size-Exclusion Chromatography (SEC)). The azidation degree (21%) was calculated by elemental analysis (E.A); E.A calculated: C(%) cal. 58.94, H(%) cal. 9.61, N(%) cal. 3.36 and E.A. found: C(%) exp. 58.30, H(%) exp. 9.21, N(%) exp. 3.62.

**Figure 1:**
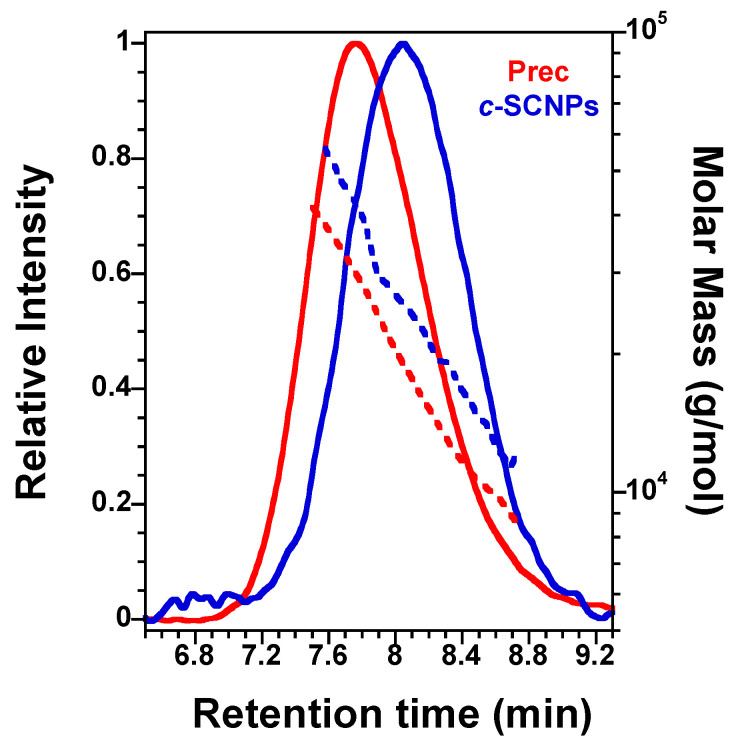
SEC chromatograms of Prec and *c*-SCNPs showing the shift of the SEC retention peak (solid lines) and molar mass of Prec upon chain compaction via “click” chemistry to produce *c*-SCNPs (dashed lines).

Synthesis of Click Single-Chain Nano-Particles (*c*-SCNPs): SCNPs were prepared via copper (I)-catalyzed azide alkyne cycloaddition (CuAAc) “click” reaction. CuBr (0.86 g, 6 mmol) and (+)-sodium l-ascorbate (1.190 g, 6 mmol) were added into a round-bottom flask of 250 mL and flushed with argon (Ar) for 15 min. Then, 130 mL of dried and degassed CH_2_Cl_2_ was added under Ar into the flask following by the addition of PMDETA (1.26 mL, 6 mmol). Finally, a solution of 150 mg of Prec in (30 mL of dried and degassed CH_2_Cl_2_ was added into the mixture to get a solution of 1 mg/mL. A solution of propargyl ether (7 µL, 0.072 mmol) in 7 mL of dried and degassed CH_2_Cl_2_ was injected to the mixture with a syringe pump at 7 mL/h. After the complete addition of the cross-linker solution, the reaction was stirred at r.t. for 24 h. The cooper catalyst was removed by extraction with a saturated solution of NH_4_Cl (4 × 100 mL). The organic phase was dried with anhydrous MgSO_4_, filtrated and evaporated at reduced pressure to obtain *c*-SCNPs as greenish viscous liquids (126 mg, 80% yield). To confirm that the SCNPs are formed, SEC analysis was carried out and was compared with the precursor (Figure 1).

Sample preparation: first either a precursor or a SCNPs solution with 2 mL of CH_2_Cl_2_ was stirred until total dissolution. Then, the mixture was drop-casted onto the aluminum flat holders used for neutron scattering experiments. Afterwards, a fume hood was used to slowly evaporate the solvent and finally under vacuum conditions the samples were well-dried in an oven at 343 K for 24 h.

Size-Exclusion Chromatography (SEC): an Agilent 1200 system equipped with PLgel 5 μm Guard and PLgel 5 μm MIXED-C columns (Santa Clara, CA, USA), a triple detection: a differential refractive index (RI) detector (Optilab Rex, Wyatt, Santa Barbara, CA, USA), a multi-angle laser light scattering (MALLS) detector (MiniDawn Treos, Wyatt, Santa Barbara, CA, USA), and a viscosimetric (VIS) detector (ViscoStar-II, Wyatt, Santa Barbara, CA, USA) were used to perform SEC measurements at temperature of 303 K. Moreover, ASTRA Software (version 6.1) from Wyatt Technology Corporation, Santa Barbara, CA, USA, was used to perform all the data analysis derived from SEC experiments. THF was used as eluent at a flow rate of 1 mL/min. A value of dn/dc = 0.062 was used for precursor and SCNP samples.

### 2.3. Thermal Analysis

The calorimetric glass transition temperature (*T_g_*) of the samples was measured using a differential scanning calorimeter (DSC) TA instrument Q2000 (TA Instruments, New Castle, DE, USA) under ultrapure nitrogen flow. In all the experiments, the sample mass used was around 5 mg. For the experiments, first, non-isothermal sweeps were performed, where the samples were heated up until 350 K. The samples were then kept at this temperature for 3 min in order to erase any previous thermal history, and finally, a cooling scan at 20 K/min was recorded to 170 K, followed by a subsequent heating scan also at 20 K/min from this temperature to 350 K. The *T_g_* values were extracted from the onset value of the jump in heat capacity.

### 2.4. Mechanical Analysis

Using an ARES rheometer (TA Instruments, New Castle, DE, USA) with a parallel plate 8 mm diameter geometry and a gap distance of ≈0.4–0.5 mm, rheological experiments in the linear regime in the frequency range of ≈0.03–16 Hz were performed. To ensure a linear regime during the experiments, a strain amplitude of 2% was used. All the experiments were carried out under a N_2_ atmosphere, and the temperature was set by an LN2 controller. The temperature range measured was 213 ≤ *T* ≤ 313 K for all the samples studied. It was observed that the principal effect of changing the temperature is to rescale the time: Temperature changes shift the viscoelastic functions along the modulus and time (or frequency) scales without changing their shapes. A result of the foregoing observation—known as time-temperature superposition (TTS)—a composite curve called a “master curve” could be generated from a series of curves of overlapping data collected at different temperatures [18].

### 2.5. Broadband Dielectric Spectroscopy (BDS)

The complex dielectric permittivity ε* (ω) = ε′ (ω) − iε″ (ω), where ε′ is the real part and ε″ is the imaginary part, was obtained as a function of the frequency ω and temperature T by using a Novocontrol high resolution dielectric analyzer (Alpha-N analyzer) (Novocontrol, Montabaur, Germany). The sample cell was set in a cryostat, and its temperature was controlled via a nitrogen gas jet stream coupled with a Novocontrol Quatro controller. Using a constant temperature, frequency scans were studied where the stability used was better than 0.05 K. The dielectric measurements were performed at different temperatures in the range of 120–310 K and at frequencies in the range of 10^−1^ to 10^7^ Hz. Measurements were carried out in the usual parallel plate geometry with electrodes of 10 and 20 mm in diameter. A separation of 0.1 mm between both electrodes was maintained by using a cross shaped Teflon^®^ spacer of small area.

### 2.6. Structural Analysis

Wide angle X-ray scattering (WAXS) technique on a Bruker D8 Advance diffractometer (Bruker, Bremen, Germany) equipment was used to study the short-range order of the systems. The equipment works in parallel beam geometry with Cu K_α_ transition photons of wavelength λ = 1.54 Å, in reflection mode (θ − 2θ configuration), varying the scattering angle 2θ from 10° to 30° with steps of 0.05°. All the measurements were performed at room temperature and the scattered intensities are shown as a function of momentum transfer *Q*, *Q* = 4π*λ*^−1^ sin θ. 

### 2.7. Quasi Elastic Neutron Scattering (QENS) Analysis

Two different spectrometers were combined to perform QENS experiments: the backscattering (BS) IN16B spectrometer and the time-of-flight (ToF) IN5 instrument, both located at ILL in Grenoble, France [19]. The finally covered *Q*-range was from 0.19 to 1.90 Å^−1^. In these experiments, three different temperatures were explored, 285, 320 and 360 K. Flat aluminum cells were used as a sample holder, with a sample thickness calculated for reaching around 90% transmission. An incident wavelength λ of 6.271 Å was used for IN16B, while λ = 5 Å was employed for IN5. The determination of the resolution function R (*Q*,*ω*) was performed by measurements at 2 K. The analysis of the quasielastic spectra was done by Fourier transforming the data to the time domain and then deconvoluting from the instrumental resolution to obtain the intermediate scattering function in the time domain *S*(*Q,t*).

## 3. Results

In order to confirm the successful formation of *c*-SCNPs from Prec via a “click” chemistry reaction prior to melt sample preparation, we show in Figure 1 how the *c*-SCNPs obtained have longer retention time at the SEC peak maximum compared to their precursor. At the same time, the *c*-SCNPs obtained were unimolecular, as the molar mass distribution remained constant. In other words, we observe a significant reduction in average hydrodynamic radius upon SCNPs formation (longer SEC retention time at peak maximum), the absence of inter-particle coupling events (no additional peaks at short SEC retention time), and similar values of the weight average molecular weight of the SCNP and precursor samples (taken at the SEC retention time corresponding to the peak maximum in each case).

**Figure 2:**
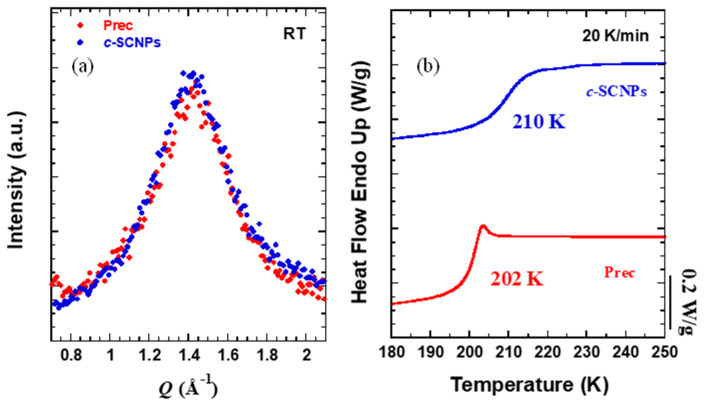
(**a**) WAXS diffraction patterns determined at room temperature (the intensity has been scaled to match at the maximum) and (**b**) heating DSC scans at 20 K/min.

WAXS experiments on the bulk samples showed that there are no appreciable differences on the short-range order of the samples. One main peak is observed in Figure 2a in the *Q*-range studied between 1.0 and 2.0 Å^−1^ centered around 1.4 Å^−1^. This peak is associated with the inter-main chain correlations and average distances of about *d_chain_* = 2π/*Q_max_* ≈ 4.4 Å [20]. The formation of SCNPs via “click” chemistry process adding internal cross-links does not affect the position of this peak.

Before delving into the microscopic relaxation, we present the macroscopic properties of the bulk *c*-SCNPs compared to its linear precursor. DSC characterization reveals the *T_g_* values of Prec and *c*-SCNPs samples in Figure 2b. The result for the precursor melt was 202 K, while for its *c*-SCNPs there is an increase of this value up to 210 K. This increase in *T_g_* suggests, for a given temperature, slower motions at the segmental level in the SCNPs.

**Figure 3:**
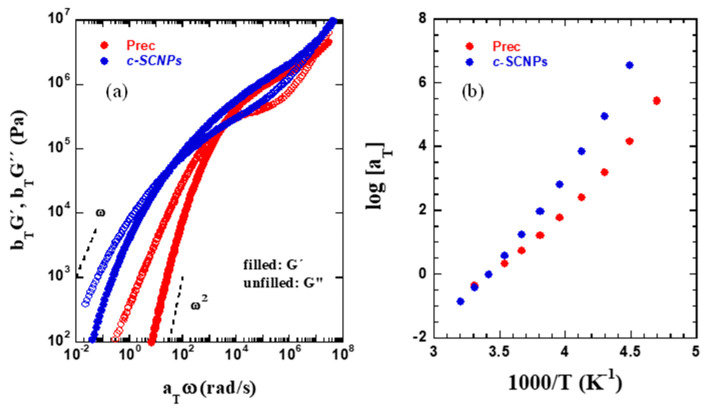
(**a**) Rheological master curves (reference temperature: 293 K) for precursor and *c*-SCNPs, built by using the shift factors shown in (**b**).

The results on the mechanical response of the precursor and its SCNPs can be seen in Figure 3a. There, the master curves obtained for the real and imaginary parts of the shear modulus of the melts are displayed. Master curves result from applying the TTS principle to isothermal curves of *G*′ (*ω*) and *G*″ (*ω*) over a broad frequency domain using the reference temperature (*T_ref_* = 293 K) [18], resulting in a smooth curve as expected for polymer melts of linear [21] and other topologies [22]. The horizontal shift factors a_T_ are shown in Figure 3b, and the vertical shift factors b_T_ ranged between 0.9 and 1.1 for all investigated samples and temperatures. Focusing first on the Prec sample, a typical linear chain polymer behavior can be observed. The curves cross at two different points: (i) one at high frequencies (about 10^7^ rad/s), where the onset of entanglement effects is reflected, and (ii) another one at low frequencies (3 × 10^3^ rad/s), where disentanglements of chains are detected. For monodisperse polymer melts below the low frequency crossing point, the moduli are expected to scale as *G*′(*ω*)~*ω*^2^ and *G*″(*ω*)~*ω*. Here, where the samples presented have polydispersity values higher than 1 (*Ð* = 1.24), the crossing point is shifted towards higher frequencies. In the case of the *c*-SCNPs sample, the cross point at high frequency occurs at similar value (around 10^7^ rad/s). The difference comes when we move to low frequencies. The crossing point is shifted to lower frequencies (3 × 10^1^ rad/s) and the transition to the terminal regime occurs at much lower frequencies. This indicates a slowing down and broadening of the structural relaxation of *c*-SCNPs compared to the precursor. On the other hand, the shift factors used to construct the master curves are displayed in Figure 3b. They show clearly a different temperature dependence towards lower temperatures consistent with the increase in *T_g_* measured by DSC.

**Figure 4:**
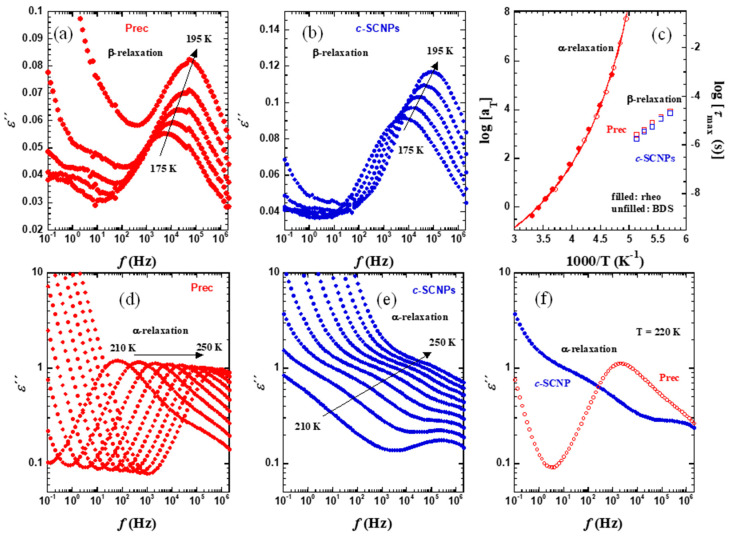
Isothermal plots of *ε*″ in the glassy state (**a**,**b**) and in the supercooled liquid state (**d**,**e**) for Prec sample (**a**,**d**) and *c*-SCNPs sample (**b**,**e**). The characteristic times obtained from the maxima of the loss peaks are represented in (**c**) (β-relaxation: unfilled squares; α-relaxation: unfilled circles). For Prec sample, the rheological shift factors are also shown for comparison (filled circles). The solid line is the VFT fit of BDS Prec results. In (**f**), a direct comparison of the results from the two samples at *T* = 220 K is shown.

BDS results (imaginary part of the permittivity) as a function of frequency are shown in the isothermal plots of Figure 4a,b,d–f. Relaxation processes appear as peaks in the dielectric loss spectra, moving toward higher frequencies as temperature increases.

For comparison, different temperature intervals are plotted. In Figure 4a,b, the temperature range chosen is from 175 and 195 K in 5 K steps, i.e., in the glassy state of both samples. The relaxation observed corresponds to the secondary *β*-process, which is in general attributed to short-range motions of lateral or short chain segments [23]. To characterize the *β*-relaxation process, the relaxation time from each isothermal plot has been calculated as:

(1) $$\tau_{max}={\left(2\pi f_{max}\right)}^{-1}$$

The resulting data have been plotted in Figure 4c.

In Figure 4d,e, the temperature range chosen is from 210 to 250 K for every 5 K, i.e., around and above the glass transition temperatures of the two samples. The main relaxation process observed in these figures corresponds to the so-called *α*- or segmental relaxation. In this process, the characteristic time strongly increases as the temperature decreases toward the glass transition. For the *c*-SCNPs sample, the strong contribution from the conductivity at low frequencies attributed to the presence of salts involved during the synthesis process prevents an accurate determination of the characteristic times of the *α*-relaxation. Nevertheless, from direct comparison of the results at a given temperature, as shown in Figure 4f, it can be seen that the α-relaxation peak of the *c*-SCNPs is centered at lower frequencies than that of the precursor sample, being consistent with an observed shift in *T_g_* as observed by DSC. For the Prec sample, Figure 4c includes also the characteristic times for the α-relaxation defined from the maximum (Equation (1)). This figure also shows the comparison of the temperature-dependent rheological shift factors with these dielectric results. A very good agreement is found between both sets of data. The fact that the rheological TTS shift factors have similar temperature dependence as the α-relaxation time confirms the thermorheological simplicity of the system. The dielectric results have been fitted using a Vogel-Fulcher-Tamman (VFT) equation [24,25,26]:

(2) $$\tau \left(T\right)=\tau_{\infty }\exp\left[\frac{B}{T-T_{0}}\right]$$

Here *T*_0_ is the Vogel temperature, *B* is an energetic term, and τ_∞_ corresponds to a typical vibration frequency. The values found were log[τ_∞_(s)] = −12.285 ± 0.28, *T*_0_ = 158 ± 2 K and *B* = 1167 ± 86 K.

**Figure 5:**
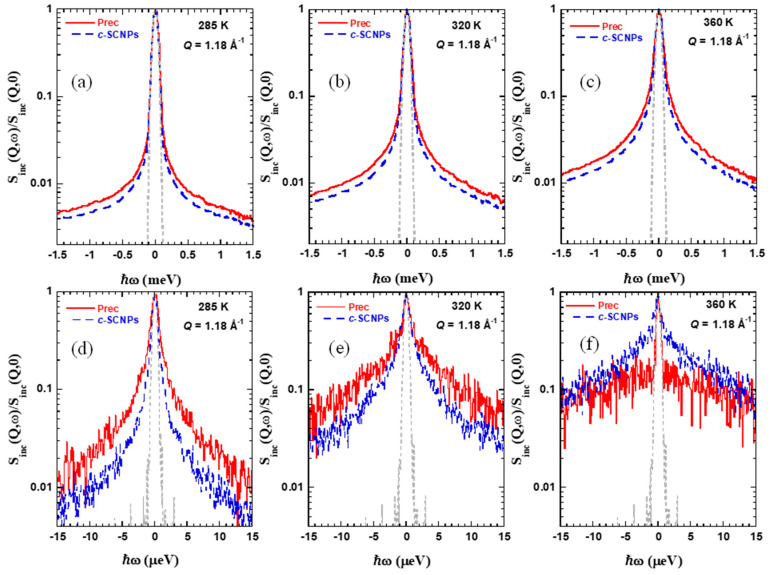
Normalized IN5 (**a**–**c**) and IN16B (**d**–**f**) spectra obtained at *Q* = 1.18 Å^−1^ and the different temperatures indicated for Prec (solid line) and *c*-SCNPs (dashed line). The dotted line shows the instrumental resolution function.

Particularly important for our investigation is the microscopic insight provided by QENS. This technique allows exploring the dynamics in the high-frequency range with spatial resolution. In QENS experiments, the number of neutrons scattered which have experienced a change in energy between ℏω and ℏω + *d*ℏω, relative to the number of incident neutrons, is measured as function of the scattering vector *Q* [27,28]. This magnitude depends on the interactions of neutrons with the atomic nuclei in the sample [28]. These interactions are characterized by the scattering length *b_α_*, where this parameter is different for different isotopes of a given element *α*, and also for different nuclear spin states. In addition, the measured cross section is determined by the (time dependent) distribution of scattering centers in the sample. In protonated samples, like those investigated in this work, the overwhelming contribution to the measured scattered intensity is the correspondence to incoherent scattering from the hydrogens, revealing correlations between the position of a given proton at different times, averaged over all the hydrogens in the sample. Figure 5 shows some representative examples of the dynamic structure factor measured in the frequency domain, normalized to its value at ℏω = 0, in order to compare directly the data obtained from these experiments. The chosen *Q*-value is 1.18 Å^−1^; results at the three different T-values studied are shown. A clear broadening with respect to the resolution function can be observed for all samples and temperatures measured. The width of a quasielastic spectrum is related with the inverse of the characteristic time of motion probed by the instrument. From this direct comparison, already a difference is observed between the Prec and *c*-SCNP samples. We note that, at the highest temperature investigated, the spectra are extremely broad in the IN16B window (Figure 5f) and that the comparison by normalization of the data in the elastic peak region is very sensitive to the background subtraction. Taking this into account, we can state that, in general, the spectra corresponding to the *c*-SCNPs sample are narrower for all temperatures, suggesting a slower dynamics in both dynamic windows explored as compared to its linear precursor sample.

**Figure 6:**
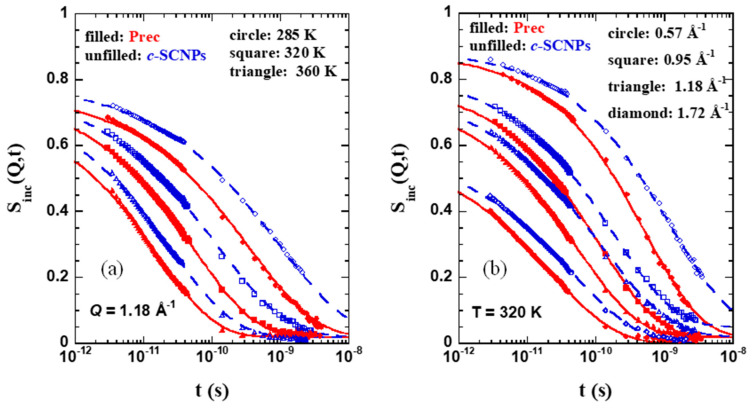
Fourier transformed and deconvoluted QENS spectra obtained from IN5 and IN16B on the precursor (red) and *c*-SCNP (blue) samples at (**a**) three different temperatures investigated: 285 K (circle), 320 K (square), and 360 K (triangle) and a *Q*-value of 1.18 Å^−1^; and (**b**) for T = 320 K at four different *Q*-values: 0.57 Å^−1^ (circle), 0.95 Å^−1^ (square), 1.18 Å^−1^ (triangle), and 1.72 Å^−1^ (diamond). Solid lines are KWW fits with β ≈ 0.5 (exact values shown in Figure 7b) to the results above 2 ps.

The spectra measured in the frequency domain are affected by instrumental resolution through convolution. Therefore, the analysis of the quasielastic spectra was done by Fourier transforming the data to the time domain and then deconvoluting from the instrumental resolution to obtain the intermediate incoherent scattering function in the time domain *S_inc_*(*Q,t*). Some representative examples are shown in Figure 6. Figure 6a corresponds to the analysis done at a *Q*-value of 1.18 Å^−1^ and three different T-values, and Figure 6b considers a fixed T-value of 320 K (the intermediate T investigated) and four representative *Q*-values explored. 

**Figure 7:**
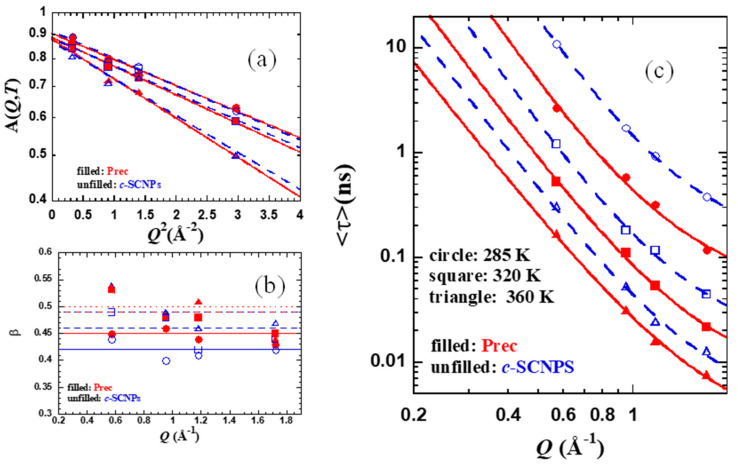
Scattering vector dependence of the parameters obtained from KWW fits (Equation (3)) to the Prec (filled red symbols) and *c*-SCNPs (empty blue symbols) QENS data at 285 K (circles), 320 K (squares), and 360 K (triangles). (**a**) Prefactors A on a log scale versus *Q*^2^. Lines are fits of a Debye–Waller-Factor like expression (solid: Prec; dashed: c-SCNPs) (**b**) stretching parameter. Horizontal lines show the average value for each temperature (solid: 285 K; dashed: 320 K; dotted: 360 K) and each sample in the corresponding color, and (**c**) average characteristic time $\langle \tau \rangle =\tau_{s}\Gamma \left(1/\beta \right)/\beta$ for H-self motions obtained for Prec (filled symbols) and *c*-SCNPs (empty symbols) at 285 K (circles), 320 K (squares), and 360 K (diamonds), imposing a fixed β-value (Figure 7b and Table 1) for each fitting. Lines are fits of Equation (4) to the Prec (solid lines) and *c*-SCNPs (dashed lines).

The QENS results can be well described above ≈2 ps by means of Kohlrausch-Williams-Watts (KWW) or stretched exponential functions

(3) $$S_{inc}\;\left(Q,t\right)=A\left(Q\right)\exp\left[-{\left(\frac{t}{\tau_{s}}\right)}^{\beta }\right]$$

with a stretching exponent β close to 0.5. The prefactor A(Q) accounts for the decay of the correlation function at shorter times due to vibrational and other fast contributions. A small elastic contribution was also allowed in the fits, which had a value of ≈0.02, and <0.05 in all cases. This component is able to account for an inaccurate subtraction of the background in the experiment. As observed in Figure 6, the description works well for both series of samples in the covered *Q*-range. The compilation of the results obtained for the prefactor A(Q), the β parameter, and the average characteristic times are shown in Figure 7a–c, respectively. In the case of a KWW function, as expressed in Equation (3), the average characteristic time is given by $\langle \tau \rangle =\tau_{s}\Gamma \left(1/\beta \right)/\beta$.

The prefactor values could be well described, as seen in Figure 7a, by a Debye–Waller-Factor like expression A(Q) ≈ exp[−<u^2^>Q^2^/3]. The values obtained for the mean squared displacement in the fast regime <u^2^> can be found in Table 1. They increase with temperature and are slightly larger for the hydrogens in the Prec sample. The same occurs with the values of the β-parameter averaged over the *Q*-values explored, indicated by the horizontal lines in Figure 7b and listed in Table 1. 

As was observed before in glass-forming systems [13,29,30], in the low *Q*-range explored, the characteristic time for H-self motions was found to follow a power law <τ> ∝ *Q^−^*^2/β^ as dictated by the Gaussian prediction. At high *Q*s, deviations from this Gaussian behavior manifest. In jump diffusion models, finite jump lengths tend to cause a bending of the dispersion for the diffusive relaxation times away from the *Q*^−2^ law, which is valid for simple diffusion at low *Q*. Based on this explanation, the generalization by the anomalous jump diffusion (AJD) model to the case of subdiffusive behavior predicts the characteristic time for the self-correlation function as

(4) $$\tau_{s}\;\left(Q\right)=\tau_{s,0}\;{\left[1+\frac{1}{Q^{2}l_{0}^{2}}\right]}^{\frac{1}{\beta }}$$

where *l*_0_ is the preferred jump length and τ_s,0_ is the time between jumps [30]. This model was applied to the results on the two samples investigated, using the average values of the stretching parameter listed in Table 1 for each temperature. The values obtained for the parameters involved in the model are listed in Table 1.

**Figure 8:**
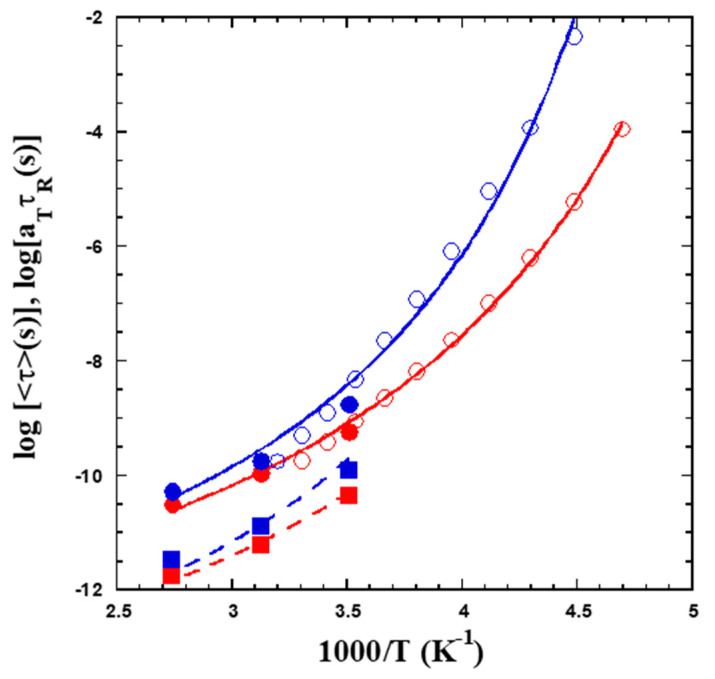
Temperature dependence of the average characteristic times obtained by QENS for a representative *Q*-value of 0.95 Å^−1^ (filled circles) and resulting values of τ_s,0_ (filled squares). The former have been combined with the corresponding rheological results (empty circles). Lines are fits of VFT functions (see the text).

Figure 8 explores the temperature dependence of the characteristic times observed by QENS. As a representative value, we have chosen *Q* = 0.95 Å^−1^. These data are compared with the rheological shift factors obtained on both samples (Figure 3b). The latter have been multiplied by suitable factors τ_R_ to coincide with the QENS results in the overlapping temperature range. For the Prec sample, the combined sets of data have then been fitted to a VFT function with the same B and *T*_0_ parameters as obtained from the BDS relaxation data. The prefactor value obtained now is log[τ_∞_(s)] = −13.06 ± 0.03. Fixing this prefactor value for the *c*-SCNPs, the fit of a VFT function yields B = 1152 ± 46 K, *T*_0_ = 178 ± 3 K. Thus, the *T*_0_ values deduced for both samples differ by 20 K. The temperature dependence displayed by the values of τ_s,0_ from the AJD model is very similar to that shown by the characteristic times at *Q* = 0.95 Å^−1^, as can be deduced from the good description of the data provided by the corresponding VFT laws properly shifted, as shown in the figure. They have been determined by keeping the same B and *T*_0_ values as used for *Q* = 0.95 Å^−1^ and fitting the prefactors, yielding log[τ_∞_(s)] = −14.29 ± 0.05 and −14.38 ± 0.13 for Prec and *c*-SCNPs respectively.

## 4. Discussion

By combining different techniques, it is possible to characterize the behavior of the different relaxations in these bulky systems and determine how they are affected by the presence of internal cross-links. We have shown that the average inter-molecular distances (position of the amorphous halo) are not appreciably influenced by the incorporation of propargyl ether cross-links. However, the dynamics of the α-relaxation is clearly modified. First, the glass-transition temperature measured by calorimetric experiments is increased (8 K) in the *c*-SCNPs sample compared to its linear polymer. Dielectric spectroscopy also shows a clear slowing down of the segmental motions involved in the α-relaxation (Figure 4), although, unfortunately, in this particular case, conductivity contributions prevent a precise quantification of this effect.

The microscopic insight provided by QENS experiments shows that the atomic motions governing the decay of the intermediate scattering function at temperatures well above the glass-transition, i.e., dominated by the segmental relaxation, are slowed down in the presence of these internal cross-links. In addition, the stretching exponent of the scattering function decreases with respect to that of the precursors, suggesting the emergence of heterogeneities in the sample induced by the cross-links. This would be the reason for a more stretched functional form of the structure factor. Moreover, the increase of the jump length with respect to that found for the Prec sample accounts for stronger deviations from Gaussian behavior in the *c*-SCNPs sample. We note that thanks to the combination of the different techniques, in particular of QENS and rheological experiments, it has been possible to determine the temperature dependence of segmental relaxation in a very wide range. The differences seen between the two samples are consistent with a shift, as observed by DSC.

The impact of propargyl ether cross-links observed on the α-process in the present bulk of protonated *c*-SCNPs is similar to that found for the melt of deuterated SCNPs synthesized following the same chemistry route [14]. In that case, an increase of 5 K was reported for the glass-transition temperature of the SCNPs with respect to the Prec counterpart. For such samples, the neutron scattering experiments were sensitive to another kind of correlation function, namely the collective dynamic structure factor. That study revealed a slowing down of the coherent scattering function at scattering vectors corresponding to the inter-molecular peak—addressing thereby the structural relaxation at a microscopic level. We note that, in the same way as in this study on the protonated *c*-SCNP samples, the average inter-molecular distance in deuterated *c*-SCNPs was unaffected by the internal cross-links. 

No effect on the segmental relaxation, however, was found on melts of (both protonated and deuterated) SCNPs obtained via the intra-molecular azide photodecomposition process involving exposition to UV irradiation [12,13]. In such a case, different monomers along the chain are directly linked through a covalent bond. Seemingly, the presence of relatively bulky moieties bridging the chain internally has a high impact in the segmental dynamics, even if the number of such cross-links is relatively small (<10%). 

Contrary to the α-process, faster dynamics and processes implying small atomic displacements seem to be hardly affected by internal cross-links. This is the case of the dielectric β-relaxation (Figure 4a–c). Such behavior was also found in the melts of SCNPs obtained by UV-irradiation [12]; therefore, it seems to be a result independent of the nature of the cross-links. Moreover, the spatial extent of the atomic (H) displacements in the subpicosecond regime as observed by QENS (see Table 1) is only subtly reduced with respect to that observed for the linear precursor counterparts at the same temperature.

At the other extreme, the structural properties and the dynamics governing the relaxation at intermediate length scales seem to be strongly affected by the topological changes induced by SCNPs formation, as was established in the previous works for both deuterated *c*-SCNPs [14] and UV-SCNPs [12,13]. For the latter, an additional relaxation process was detected by dielectric spectroscopy at lower frequencies than the α-peak, which was tentatively related to the heterogeneities provoked by the internal multiloop topology of the SCNPs and the segregation of their internal domains. We would expect that such a process would also be present in the *c*-SCNPs investigated in this work; the strong conductivity contribution, however, prevents discerning its possible existence. 

Finally, the rheological response shows a clear impact of the cross-links. The melt of *c*-SCNPs exhibits a slowing down and a broadening of the structural relaxation compared to the linear precursor. The observed effect here is very different from that reported for the deuterated counterparts, where a gel-like behavior was found. It also differs from the apparent disappearing of entanglements in the UV-SCNP samples, where the disentanglement frequency was shifted to higher frequencies with respect to the case of the precursor melt. Compared to all other relaxation techniques, stress relaxation is complex because it has contributions from dynamical processes of all polymer chain scales, from the segmental level to the entire chain. In this work, as well as the previously reported cases, there was a good agreement in the fast dynamics across all relaxation techniques including rheology. However, the long-time rheological response due to slower dynamics at the chain relaxation level differs depending on the preparation method. We may attribute this effect to the presence of residual amounts of cross-linkers or other impurities. Thus, purification efforts should be made to accurately probe the effect of the internal cross-linking on the rheology. 

## 5. Conclusions

We have investigated the impact of inducing internal bonds involving bulky cross-links on the properties of polymer melts. The application of different complementary techniques has been essential to elucidate the effects on diverse dynamical processes taking place in this kind of material. In particular, the use of QENS has allowed gaining microscopic insight into the proton displacements in the supercooled liquid regime.

Internal cross-links do not appreciably alter local properties or fast dynamics. This is the case of the average inter-molecular distances as observed by WAXS, the β-relaxation as measured by dielectric spectroscopy, and the extent of the atomic displacements at timescales faster than some picoseconds as detected by QENS. These observations were also made in the case of SCNPs synthesized by another route where no bulky cross-linkers are internally bonding the chain, suggesting that this conclusion could be extensible to melts of SCNPs in general, independently of the nature of the cross-linking agent.

A strong effect has been found on the dynamics of the α-relaxation, manifested by a slowing down of the characteristic times in the SCNPs with respect to the precursor counterparts detected by dielectric spectroscopy and QENS. This technique has also allowed to resolve broader response functions and stronger deviations from Gaussian behavior in the SCNPs melt than in the precursor. These findings could be interpreted as signatures of the presence of additional heterogeneities. Their existence, impacting mainly the intermediate length scales region, was put forward by previous SANS experiments on melts of the deuterated SCNPs. The slower motions involved in the segmental relaxation of the SCNPs lead to a shift of the glass-transition temperature of the SCNPs toward higher temperatures with respect to that in the precursor melt as observed by DSC and is also manifested in BDS and rheological experiments. We note that these effects were not reported in melts of SCNPs synthesized by UV irradiation, while a slowing down of the structural relaxation was also found on the deuterated SCNPs involving the same propargyl ether cross-links. In all cases, the molecular weights of the involved macromolecules are above the limit below which the segmental relaxation and the glass-transition temperature vary with molecular weight for the linear polymers; thus, this ingredient can be ruled out as the origin of the observed different impact of cross-links on segmental relaxation. The effects here found on the α-process might be attributed to the presence of bulky moieties bridging chain segments in the case of the SCNPs synthesized via click-chemistry (propargyl ether), which are absent in the SCNPs synthesized via UV-irradiation. In such case, direct covalent bonds connecting precursor monomers are created.

The results presented in this study, reporting the change in dynamical properties of polymeric materials when the chain topology is altered by internal bulky cross-linkers, are of great interests for practical applications of SCNPs in a variety of potential fields (e.g., all-polymer nanocomposites, enzyme-mimetic catalysis, sensing).

## Figures and Tables

**Table 1:** Values of the parameters involved in the AJD fitting the Prec and *c*-SCNPs results described with KWW functions.

	*T* (K)	<u^2^> (Å^2^)	<β>	$\tau_{s,0}^{H}\;(\mathrm{ps})$	*l*_0_ (Å)
Prec *c*-SCNPs	285	0.38	0.45	45	0.75
	285	0.40	0.42	122	0.74
Prec *c*-SCNPs	320	0.42	0.49	6.0	0.61
	320	0.40	0.46	13	0.67
Prec *c*-SCNPs	360	0.58	0.50	1.8	0.57
	360	0.54	0.49	3.4	0.62

## Data Availability

The data presented in this study are available upon request from the corresponding author.
